# Supplementary material for: Resistance of the CRISPR-Cas13a Gene-Editing System to Potato Spindle Tuber Viroid Infection in Tomato and Nicotiana benthamiana
Source: Viruses. 2024 Aug 31;16(9):1401. doi: 10.3390/v16091401 (PMC11437488; doi:10.3390/v16091401)
Supplement: Supplementary file 1 [file viruses-16-01401-s001.zip › Figure S2 Detection of PSTVd in í«Rutgersí» tomato plants using Northern hybridization.pdf]

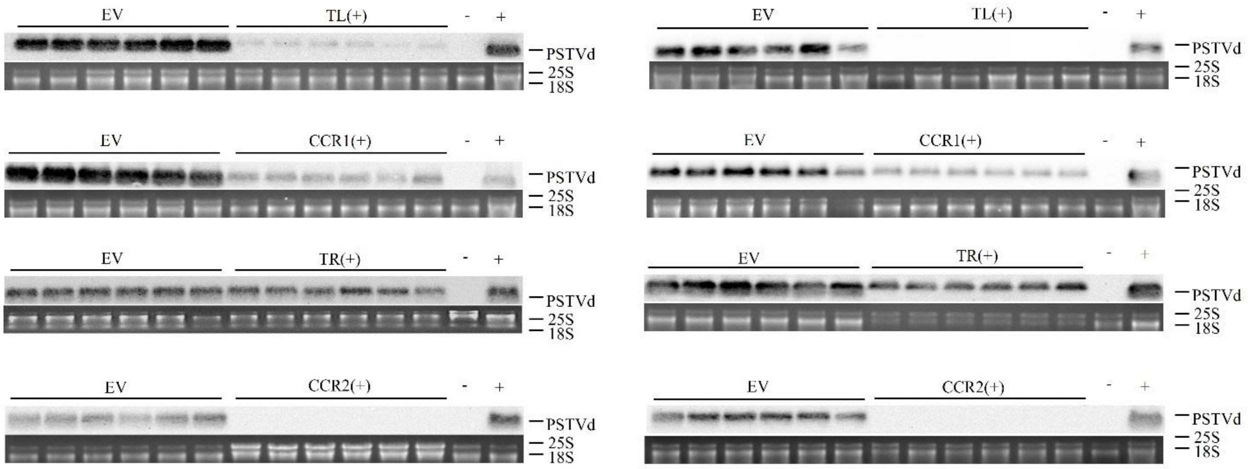

A

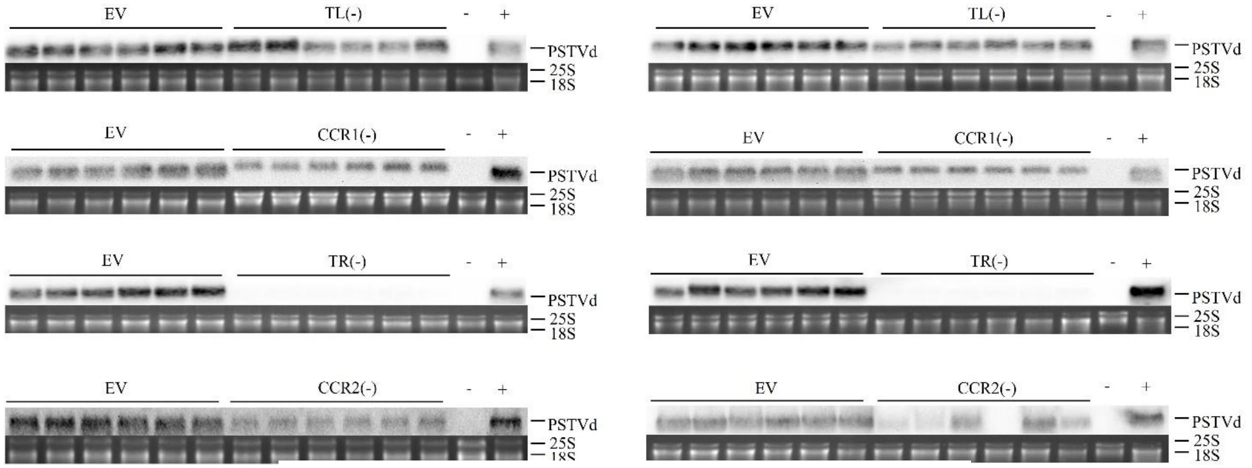

B

Figure S2. PSTVd detection in 'Rutgers' tomato plants via Northern blot hybridization. PSTVd accumulation was observed in (A) EV, TL(+), CCR1(+), TR(+), and CCR2(+) and (B) EV, TL(-), CCR1(-), TR(-), and CCR2(-) inoculated 'Rutgers' tomato plants at 30 dpi, with 18S rRNA used as the loading control. No bands were detected in TL(+) and CCR2(+) plants, while CCR1(+), TR(-), and CCR2(-) plants exhibited significantly reduced PSTVd levels. CCR1(-) and TR(+) plants displayed intermediate levels of PSTVd accumulation. TL(-) was the only construct that allowed PSTVd RNA to accumulate at levels comparable to EV plants. "-" indicates a healthy mock control, and "+" indicates a PSTVd-positive control.
