# Supplementary material for: Resistance of the CRISPR-Cas13a Gene-Editing System to Potato Spindle Tuber Viroid Infection in Tomato and Nicotiana benthamiana
Source: Viruses. 2024 Aug 31;16(9):1401. doi: 10.3390/v16091401 (PMC11437488; doi:10.3390/v16091401)
Supplement: Supplementary file 1 [file viruses-16-01401-s001.zip › Figure S3 Phenotype observation of transgenic Nicotiana benthamiana lines expressing CCR2(+).pdf]

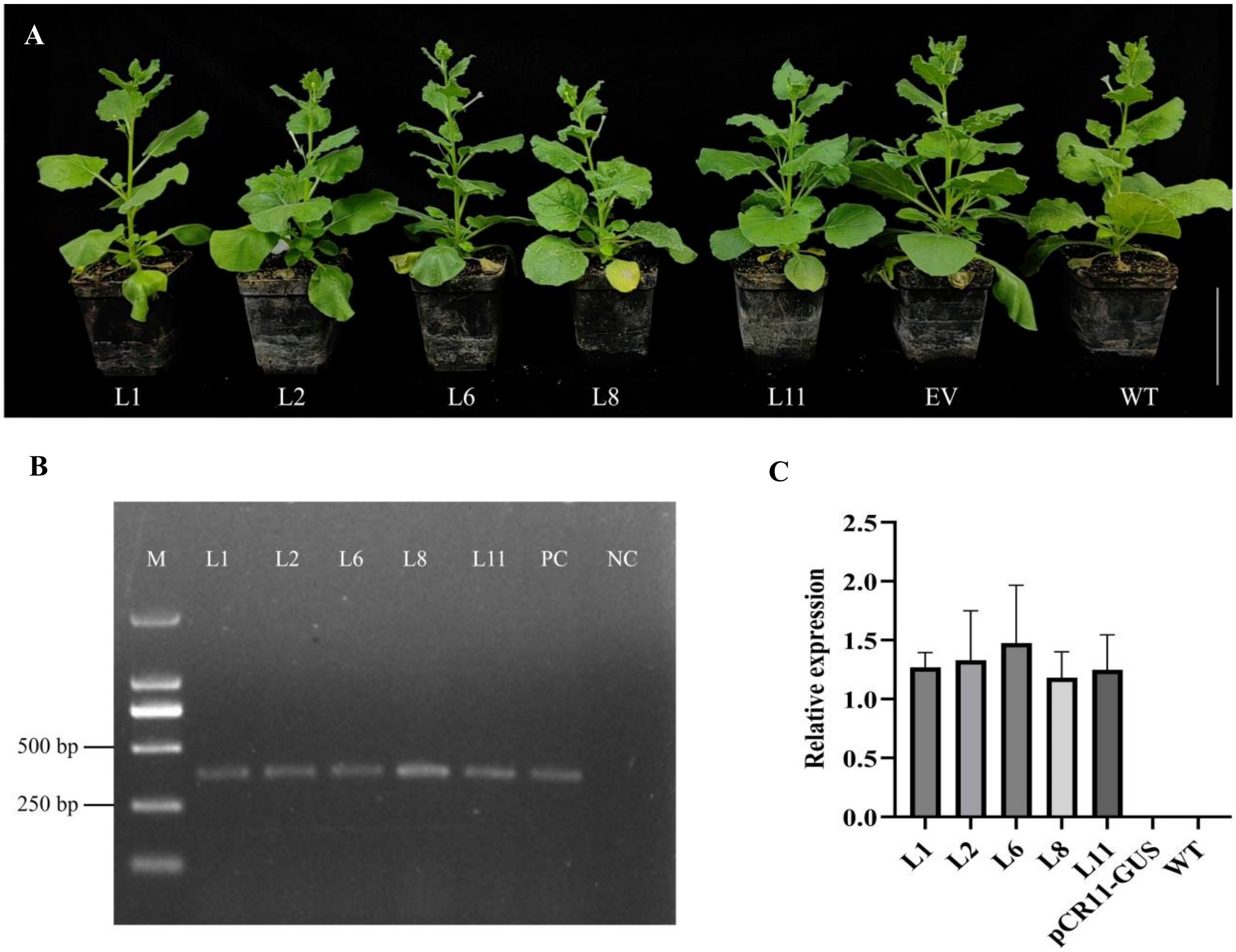

Figure S3. Phenotypic observation and Cas13a expression in transgenic *Nicotiana benthamiana* lines expressing the CCR2(+) construct, along with confirmation of its insertion. (A) Phenotypic appearance of 42-day-old wild-type (WT) *N. benthamiana* plants, pCR11-GUS (GUS replacing Cas13a), and CCR2(+) transgenic lines 1, 2, 6, 8, and 11, grown under long-day (LD; 16 h light/8 h dark) conditions in a phytotron, showing no observable phenotype differences. (B) PCR detection of the CCR2(+) construct insertion at approximately 400 bp in transgenic lines 1, 2, 6, 8, and 11 using the M13F/CCR2(+)-R primer set. (C) Cas13a expression levels in pCR11-GUS, CCR2(+) transgenic lines, and WT controls under LD conditions were assessed using reverse-transcription quantitative real-time PCR (RT-qPCR), with lines L2 and L6 showing high Cas13a expression. *Protein Phosphatase 2A* (*PP2A*) was used as the reference gene for normalization of Cas13a expression levels. Scale bar = 10 cm. M: DNA marker DL2000. Values are presented as the mean ± SE from four independent replicates (n = 4). “NC” indicates negative control, “PC” indicates positive control.
