# Supplementary material for: Resistance of the CRISPR-Cas13a Gene-Editing System to Potato Spindle Tuber Viroid Infection in Tomato and Nicotiana benthamiana
Source: Viruses. 2024 Aug 31;16(9):1401. doi: 10.3390/v16091401 (PMC11437488; doi:10.3390/v16091401)
Supplement: Supplementary file 1 [file viruses-16-01401-s001.zip › Table S1 Sequence of crRNA used in this study.pdf]

Table S1. Sequence of crRNA used in this study

Targets in PSTVd	crRNA-ID	Target sequence (5′—3′)	Oligonucleotide sequence	
TL	TL(+)	GGAACTAAACTCGTGGTTCCTGT	F：	aaacACAGGAACCACGAGTTTAGTTCC
			R：	aaaaGGAACTAAACTCGTGGTTCCTGT
C	CCR1(+)	GCGCTTCAGGGATCCCCGGGGAA	F：	aaacTTCCCCGGGGATCCCTGAAGCGC
			R：	aaaaGCGCTTCAGGGATCCCCGGGGAA
C	CCR2(+)	GCTTCGGCTACTACCCGGTGGAA	F：	aaacTTCCACCGGGTAGTAGCCGAAGC
			R：	aaaaGCTTCGGCTACTACCCGGTGGAA
TR	TR(+)	GACAGGAGTAATTCCCGCCGAAA	F：	aaacTTTCGGCGGGAATTACTCCTGTC
			R：	aaaaGACAGGAGTAATTCCCGCCGAAA
TL	TL(-)	ACAGGAACCACGAGTTTAGTTCC	F：	aaacGGAACTAAACTCGTGGTTCCTGT
			R：	aaaaACAGGAACCACGAGTTTAGTTCC
C	CCR1(-)	TTCCCCGGGGATCCCTGAAGCGC	F：	aaacGCGCTTCAGGGATCCCCGGGGAA
			R：	aaaaTTCCCCGGGGATCCCTGAAGCGC
C	CCR2(-)	TTCCACCGGGTAGTAGCCGAAGC	F：	aaacGCTTCGGCTACTACCCGGTGGAA
			R：	aaaaTTCCACCGGGTAGTAGCCGAAGC
TR	TR(-)	TTTCGGCGGGAATTACTCCTGTC	F：	aaacGACAGGAGTAATTCCCGCCGAAA
			R：	aaaaTTTCGGCGGGAATTACTCCTGTC
