# Supplementary material for: Resistance of the CRISPR-Cas13a Gene-Editing System to Potato Spindle Tuber Viroid Infection in Tomato and Nicotiana benthamiana
Source: Viruses. 2024 Aug 31;16(9):1401. doi: 10.3390/v16091401 (PMC11437488; doi:10.3390/v16091401)
Supplement: Supplementary file 1 [file viruses-16-01401-s001.zip › Table S2 List of oligonucleotides used in this study.pdf]

Table S2. List of oligonucleotides used in this study.

Oligonucleotide	Sequence 5′ to 3′	Description and Use
SK primer	TCTAGAACTAGTGGATC	Forward primer for PCR amplification of PSTVd-s insert in pC1300-PSTVd-s
PSTVd-R	GCAGCCCGGGGGATCCCC	Reverse primer for PCR amplification of PSTVd-s insert in pC1300-PSTVd-s
M13F	TGTAAAACGACGGCCAGT	Forward primer for PCR amplification of each crRNA insert in pCR11
pCR11-R	GCTCCACCATGTTGACCT	Reverse primer for PCR amplification of each crRNA insert in pCR11
Cas13a-F	CAGGAGAACGAGGAAGAGATCGA	Forward primer for RT-qPCR analysis of *Cas13a* expression
Cas13a-R	CAGTCTCGTTCTCGATGATCTTCTC	Reverse primer for RT-qPCR analysis of *Cas13a* expression
PP2A-F	GTGAAGCTGTAGGGCCTGAGC	Forward primer for RT-qPCR analysis of *PP2A* expression
PP2A-R	CATAGGCAGGCACCAAATCC	Reverse primer for RT-qPCR analysis of *PP2A* expression
M13F	CGCCAGGGTTTTCCCAGTCACGAC	Forward primer for PCR amplification of CCR2(+) insert in transgenic *N. benthamiana* plants
CCR2(+)-R	GCTTCGGCTACTACCCGGTGGAA	Reverse primer for PCR amplification of CCR2(+) insert in transgenic *N. benthamiana* plants
