# Supplementary material for: Resistance of the CRISPR-Cas13a Gene-Editing System to Potato Spindle Tuber Viroid Infection in Tomato and Nicotiana benthamiana
Source: Viruses. 2024 Aug 31;16(9):1401. doi: 10.3390/v16091401 (PMC11437488; doi:10.3390/v16091401)
Supplement: Supplementary file 1 [file Figure S1.pdf › Figure S1.pdf]

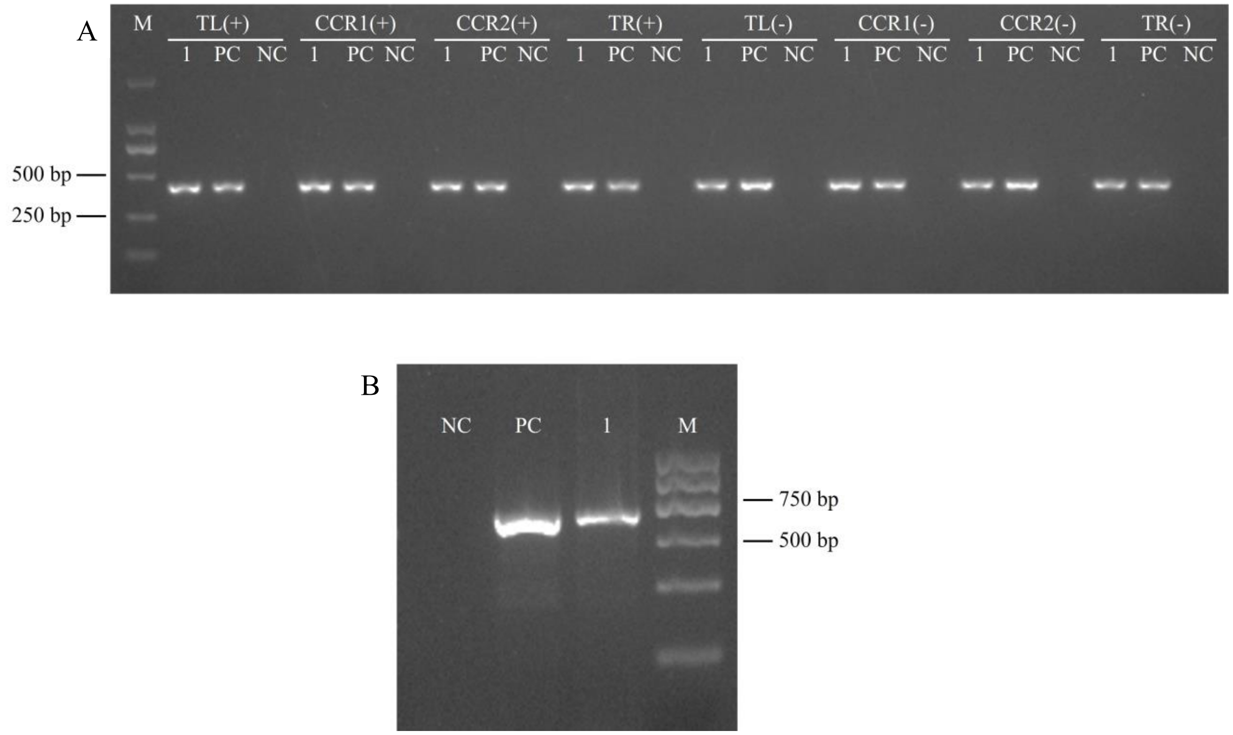

Figure S1. Detection of crRNA-Cas13a and pC1300-PSTVd-s in *Agrobacterium tumefaciens* strain *GV3101* via PCR using the M13F/pCR11-R and SK primer/PSTVd-R primer sets, respectively. (A) The crRNA sequences for TL(+), CCR1(+), TR(+), CCR2(+), TL(-), CCR1(-), TR(-), and CCR2(-) were detected at approximately 400 bp. (B) The PSTVd-s sequence was detected at approximately 700 bp. "1" denotes the detected sample; M represents the DL2000 marker; NC indicates the negative control; PC indicates the positive control.
